# CHDH, a key mitochondrial enzyme, plays a diagnostic role in metabolic disorders diseases and tumor progression

**DOI:** 10.3389/fgene.2023.1240650

**Published:** 2023-08-02

**Authors:** Yifei Li, Xinzhuang Shen, Xiaowen Yang, Fuming Lian, Yanping Li, Jinmeng Li, Yongming Huang, Wenzhi Shen, Huan Liu

**Affiliations:** ^1^ College of Clinical Medicine, Jining Medical University, Jining, China; ^2^ Key Laboratory of Precision Oncology in Universities of Shandong, Institute of Precision Medicine, Jining Medical University, Jining, China; ^3^ Department of General Surgery, Affiliated Hospital of Jining Medical University, Jining Medical University, Jining, China

**Keywords:** choline dehydrogenase (CHDH), mitochondrial enzyme, metabolic disorders diseases, tumor, biomarker, single nucleotide polymorphism (SNP)

## Abstract

Human choline dehydrogenase (CHDH) is a transmembrane protein located in mitochondria. CHDH has been shown to be one of the important catalytic enzymes that catalyze the oxidation of choline to betaine and is involved in mitochondrial autophagy after mitochondrial damage. In recent years, an increasing number of studies have focused on CHDH and found a close association with the pathogenesis of various diseases, including tumor prognosis. Here we summarized the genomic localization, protein structure and basic functions of CHDH and discuss the progress of CHDH research in metabolic disorders and other diseases. Moreover, we described the regulatory role of CHDH on the progression of different types of malignant tumors. In addition, major pathogenic mechanisms of CHDH in multiple diseases may be associated with single nucleotide polymorphism (SNP). We look forward to providing new strategies and basis for clinical diagnosis and prognosis prediction of diseases by diagnosing SNP loci of CHDH genes. Our work evaluates the feasibility of CHDH as a molecular marker relevant to the diagnosis of some metabolic disorders diseases and tumors, which may provide new targets for the treatment of related diseases and tumors.

## Introduction

Choline dehydrogenase (CHDH), acts as one of the key enzymes of choline metabolism, catalyzes the dehydrogenation of choline to betaine aldehyde in the mitochondria. Under the action of betaine aldehyde dehydrogenase (BADH), betaine aldehyde is converted to betaine, an important methyl donor for the synthesis of methionine from homocysteine (Roci et al., 2020). In terms of physiological functions, CHDH regulates the balance of free choline concentration and can participate in mitochondrial autophagy (Park et al., 2014). In recent years, the role of CHDH in medicine has attracted attention. Different studies have found that CHDH is closely associated with the development of several diseases, including malignancies (Chen et al., 2022; Wei et al., 2022; Zhang et al., 2022), psychiatric disorders (Chang et al., 2017; Truong et al., 2022), male infertility (Johnson et al., 2012; Lazaros et al., 2012), tooth agenesis (Mostowska et al., 2011) and hyperhomocysteinemia (Kumar et al., 2009). In addition, when choline is used as a therapeutic agent, CHDH may be responsible for part of the rapid turnover of free choline so that free choline does not remain elevated and inhibits choline from exerting its pharmacological effects (Haubrich and Gerber, 1981).

In this review, we will summarize the genomic localization, protein structure and basic functions of CHDH and discuss the progress of CHDH research in metabolic disorders and other diseases. Moreover, we will describe the regulatory role of CHDH on the progression of different types of malignant tumors. In addition, major pathogenic mechanisms of CHDH in multiple diseases may be associated with single nucleotide polymorphism (SNP) will also discussed. We look forward to providing new strategies and basis for clinical diagnosis and prognosis prediction of diseases by diagnosing SNP loci of CHDH genes.

### Location and structure of CHDH

**FIGURE 1:**
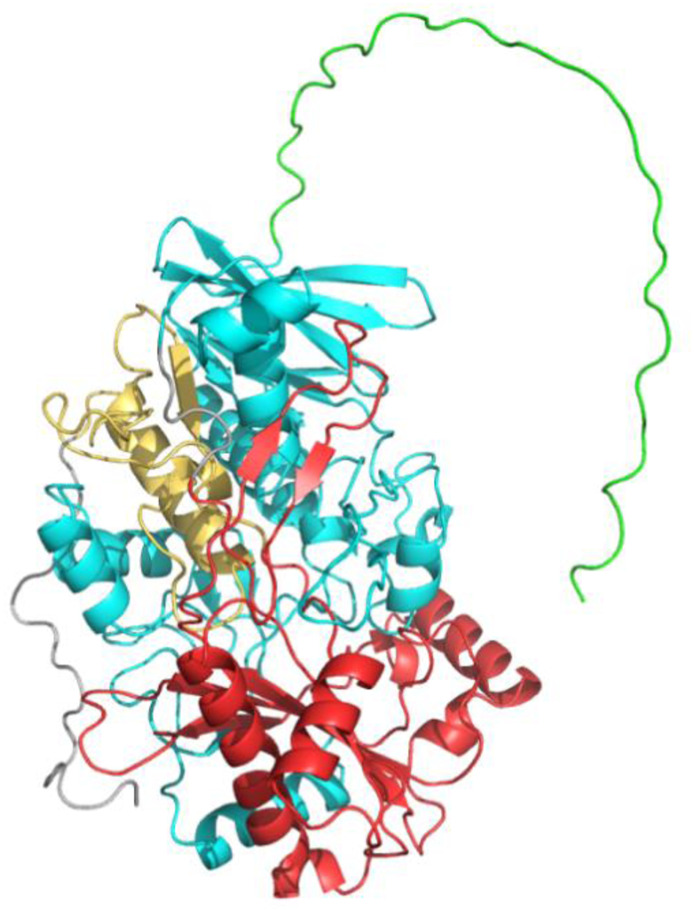
Basic structure and different structural domains of CHDH. 1 to 38: N-terminus, green; 39 to 326: FAD/NAD(P)-binding domain 1 (FB1), cyan; 333 to 510: FAD-linked reductase domain (RD), red; 511 to 574: FAD/NAD(P)-binding domain 2 (FB2), yellow.

Choline dehydrogenase (CHDH; E.C. 1.1.99.1) is a nuclear-encoded mitochondrial enzyme that is one of the key enzymes in choline metabolism. Based on NCBI gene database information, the CHDH encoding gene is located on chromosome 3 (gene location 3p21.1) with a protein molecular weight of 65KD. With the continuous development of protein technology, the protein structure of CHDH has been gradually clarified. Through the analysis and report of CHDH structure by Park et al., CHDH mainly consists of N-terminal (residues 1–38) and three functional domains, namely, flavin adenine dinucleotide (FAD)/nicotinamide adenine dinucleotide (NAD) (P)-binding domain 1 (FB1, residues 39–326), FAD-linked reductase domain (RD, residues 333–515) and FAD/NAD(P)-binding domain 2 (FB2, residues 511–574) (Figure 1). Meanwhile, the functions of each structural domain have been continuously discovered.

In the study by Haubrich et al., CHDH is found to be highly expressed in the kidneys, with only one-sixth as active in the liver as the kidneys. It is found in other tissues, such as the blood, spleen and heart, but not in fat and muscle tissue (Haubrich and Gerber, 1981). In humans, CHDH is expressed in both the inner and outer mitochondrial membranes (Park et al., 2014).

CHDH is one of the enzymes initially classified in the glucose-methanol-choline (GMC) enzyme oxidoreductase superfamily according to the primary structural arrangement (Salvi and Gadda, 2013). The GMC superfamily is a family of oxidoreductases with a common fold structure. In this family, FAD is a coenzyme that generally oxidizes alcohol substrates to aldehydes or ketones (Sutzl et al., 2019). In a study on purified rat liver CHDH, Tsuge et al. noted that “all the characteristics reported so far were obtained using relatively crude preparations, and highly purified preparations are an imperative in the field” (Tsuge et al., 1980). Due to the difficulties in purifying CHDH, biochemists lost interest in the enzyme and little is known about the biochemical properties of human CHDH. Apart from genetic studies that isolated and measured enzyme activity from tissue homogenates, there are no reports on human CHDH (Haubrich and Gerber, 1981).

### Physiological role of CHDH

**FIGURE 2:**
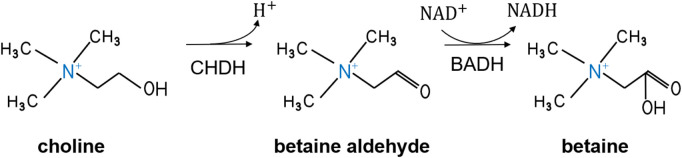
Metabolic process of choline oxidation to betaine. CHDH, choline dehydrogenase; BADH, betaine aldehyde dehydrogenase; NAD^+^, nicotinamide adenine dinucleotide; NADH, reduced nicotinamide adenine dinucleotide.

CHDH is a FAD-dependent enzyme that converts choline to betaine aldehyde. Betaine aldehyde is further oxidized to betaine and reduced nicotinamide adenine dinucleotide (NADH) by BADH (Figure 2). CHDH regulates intracellular choline and free choline concentrations (Haubrich and Gerber, 1981), and the resulting betaine has a protective effect on cellular infiltration and functions as a methyl donor (Yeroshkina et al., 2022).

**FIGURE 3:**
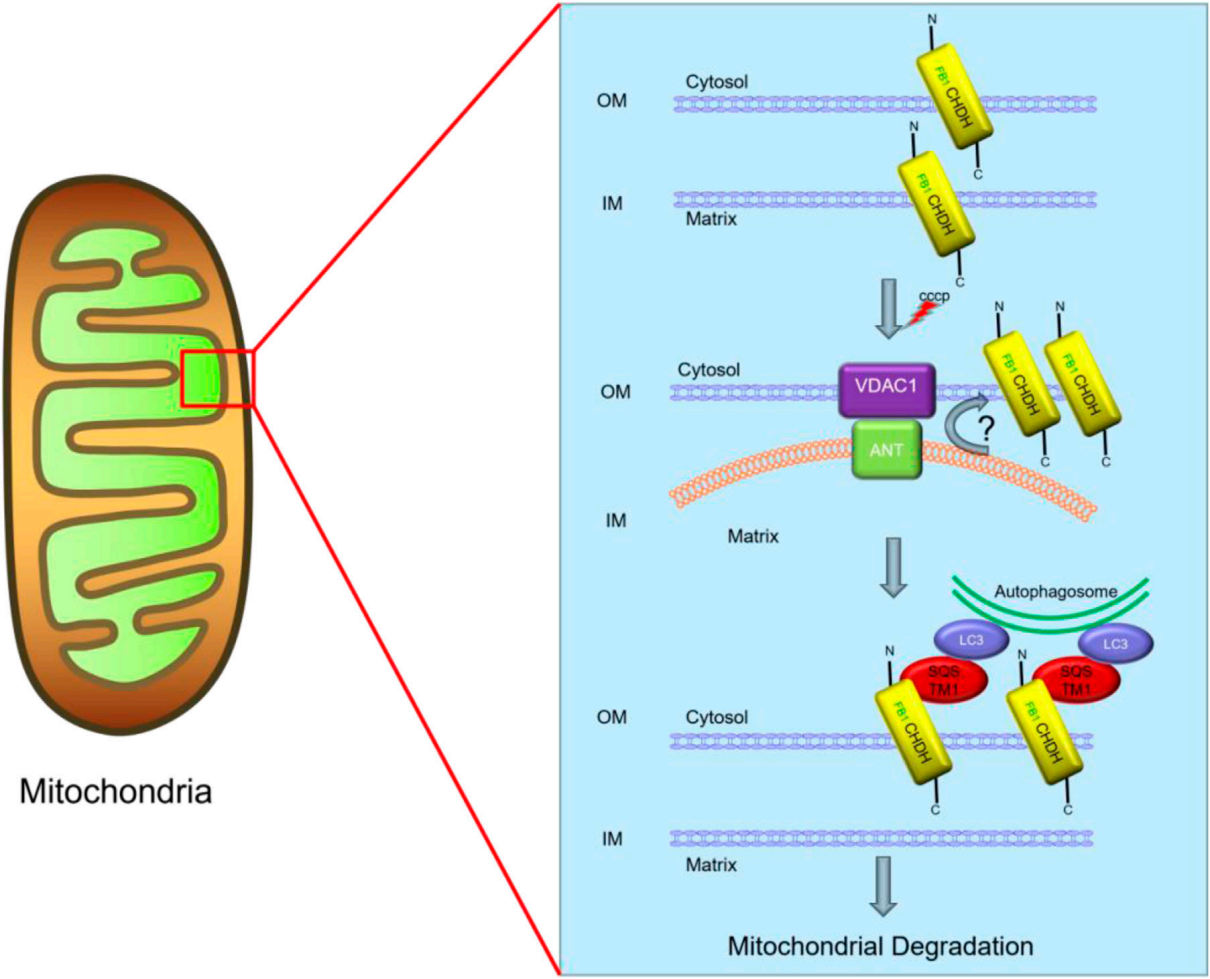
Predictive role of CHDH in mitochondrial autophagy. CCCP, carbonyl cyanide m-chlorophenylhydrazone.

CHDH also plays a key role in mitochondrial autophagy. Based on the report of Park et al., CHDH is a transmembrane protein on mitochondria that is expressed on both the outer membrane (OM) and inner membrane (IM) of mitochondria and exposes its N-terminal end containing the FB1 structural domain to the cell membrane on the OM. CHDH on the mitochondrial OM plays an important role in mitochondrial autophagy. When mitochondrial damage occurs, the membrane potential of mitochondria is altered. CHDH may accumulate on the outer mitochondrial membrane through the voltage-dependent anion channel 1 (VDAC1) on the mitochondrial membrane as a contact point in response to the altered membrane potential. The autophagic adaptor p62 (also known as SQSTM1) acts as an important adaptor linking damaged cargo to LC3, interacting with FB1, which then recruits LC3 to damaged mitochondria and stimulates mitochondrial autophagy (Figure 3) (Park et al., 2014).

As a methyl donor, betaine methylates homocysteine to methionine and produces dimethylglycine by the action of betaine homocysteine S-methyltransferase (BHMT) (Yeroshkina et al., 2022). Betaine produces dimethylglycine mainly in the liver and kidney (McKeever et al., 1991; Perez-Miguelsanz et al., 2017). This reaction is similar to the homocysteine remethylation reaction of the vitamin B12-folate pathway (Figueroa-Soto and Valenzuela-Soto, 2018). The BHMT pathway accounts for half of the hepatic methylation of homocysteine (Feng et al., 2011). Methionine is a precursor of the methyl donor S-adenosylmethionine (SAM), which is involved in various methylation reactions, such as DNA, RNA, and proteins (Struck et al., 2012). Methionine and serine are considered to be the main sources of mononuclear groups in proliferating cells. In humans and mammals, betaine enters renal medullary cells via the betaine/GABA transporter (BGT1), which plays an important role in osmoprotection (Kempson et al., 2014). In the liver, betaine regulates the hydration of hepatocytes (Hoffmann et al., 2013). However, in a recent study by Sung et al. betaine catalyzed by choline dehydrogenase in tumor cells of different tissues was not involved in methylation. Moreover, at intracellular betaine concentrations of approximately 10 μM, betaine did not provide the infiltration protection reported in other cell types (Roci et al., 2020). Therefore, the role of CHDH in producing metabolic pathway products in cancer cells needs to be further investigated.

**FIGURE 4:**
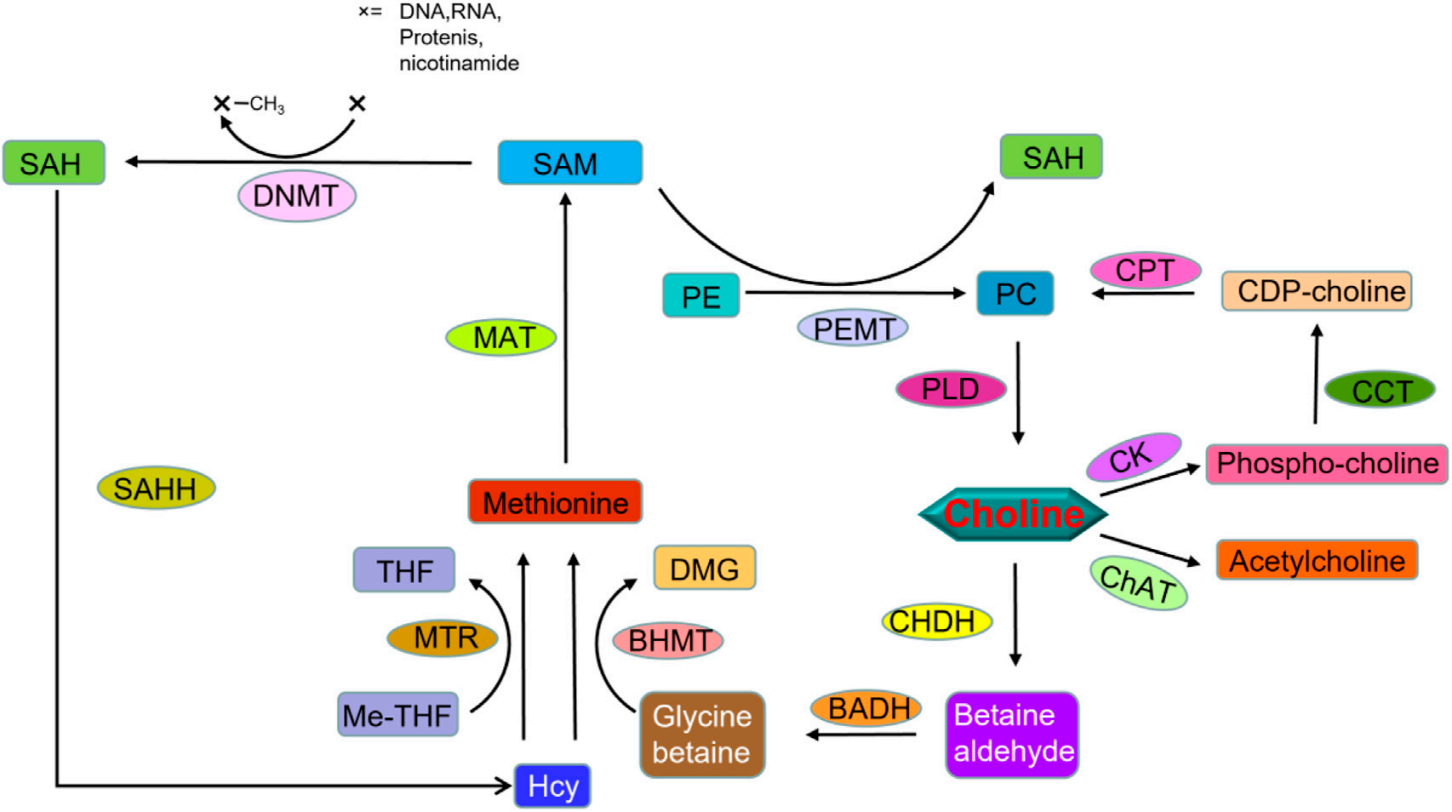
Molecular network of choline metabolism. CHDH, choline dehydrogenase; BADH, betaine aldehyde dehydrogenase; BHMT, betaine-homocysteine methyltransferase; DMG, dimethylglycine; Hcy, homocysteine; Me-THF, methyl tetrahydrofolate; THF, tetrahydrofolate; MTR, methionine synthase; MAT, methionine adenosyltransferase; SAM, S-adenosylmethionine; SAH, S-adenosylhomocysteine; DNMT, DNA (cytosine-5-)-methyltransferase; SAHH, S-adenosylhomocysteine hydrolase; CK, choline kinase; CCT, choline-phosphate cytidylyltransferase; CDP-choline, Cytidine diphosphate choline; CPT, diacylglycerol cholinephosphotransferase; PE, phosphatidylethanolamine; PC, phosphatidylcholine; PEMT, phosphatidylethanolamine N-methyltransferase; PLD, phospholipase D; ChAT, choline O-acetyltransferase.

Choline is a core nutrient for human metabolism and is important for maintaining cellular form and function, including cell membrane synthesis, neurotransmitters and epigenetic regulation of DNA (Figure 4). The most abundant lipid in cell membranes is phosphatidylcholine, so large amounts of choline are required to synthesize the membranes of proliferating cells. Choline is essential for most proliferating cells. Choline is present in almost all cell cultures at high concentrations, usually around 10–100 μM, comparable to most amino acids (Roci et al., 2020).

### Relevant role of CHDH in medical diseases

With the increasing research on CHDH in medicine, it has been found that CHDH plays an important role in the development of many diseases. The decrease or increase in CHDH due to single nucleotide polymorphism (SNP), which affects choline metabolism and turnover of the methionine-homocysteine cycle, may be the main mechanism responsible for the disease. Several SNPs of CHDH are common in humans and are expected to be prognostic assessment factors for malignancies (Table 1) and potential therapeutic targets for related diseases.

**TABLE 1:** Selected studies showing the relationship of CHDH with tumorigenesis and progression.

Tumor type	CHDH status	Tumorigenesis	Cancer progression	Ref.
Breast cancer	Low expression	-	Favorable	Ma et al. (2006); Ma et al. (2004)
Breast cancer	Mutation	Favorable	-	Xu et al. (2008)
Pancreatic cancer	Mutation	Unfavorable	-	Chittiboyina et al. (2018)
Head and neck squamous cell carcinoma	Low expression	-	Unfavorable	Wu et al. (2020)
Clear cell renal cell carcinoma	Low expression	-	Favorable	Zhang et al. (2022)
Gastric cancer	Low expression	-	Favorable	Chen et al. (2022)
Hepatocellular carcinoma (tumor-adjacent non-cancer tissues)	Overexpression	-	Favorable	Wei et al. (2022)

### CHDH and malignancy

#### Breast cancer

Breast cancer has become the world’s most common malignancy (Siegel et al., 2022). Ma et al. performed gene expression profiling in the cohort of 60 women receiving adjuvant tamoxifen alone in an early stage estrogen receptor positive breast cancer patient cohort, and found a significant difference of CHDH in genes between tamoxifen treated breast cancer patients who relapsed and did not relapse (Ma et al., 2004). In the expanded cohort study, low expression of CHDH was associated with a higher risk of recurrence after tamoxifen monotherapy in the breast cancer patient cohort (Ma et al., 2006). A study on CHDH as a prognostic biomarker for breast cancer found that its expression is regulated by estrogen and its prognostic function may be mediated by estrogen-dependent pathways (Wang et al., 2007). A population-based study proposed oxidative metabolism of choline was associated with the risk of breast cancer. Higher dietary intake of choline reduced the risk of breast cancer and the carrier minor T allele of the CHDH rs12676 (+432G>T) SNP was correlated to an increased risk of breast cancer (Xu et al., 2008).

#### Pancreatic cancer

Pancreatic cancer is recognized as the “king of cancers” (Mizrahi et al., 2020). Low folate levels are one of the risk factors for pancreatic cancer. In addition to determinants such as nutrition and lifestyle, folate levels can be influenced by genetic factors, such as SNPs. A recent study showed a significant difference in the distribution of CHDH rs12676 (+432G>T) SNP genotypes in pancreatic cancer patients compared to healthy individuals. LL genotypes with the CHDH L78R SNP showed significant protection against pancreatic cancer compared to the reference RR allele (Chittiboyina et al., 2018). Although the above studies did not delve into the effect of SNPs on CHDH enzyme activity, several studies have shown that substitution of L78R can lead to altered protein function (Domoszlai et al., 2014). CHDH promotes mitochondrial autophagy by damaging mitochondria (Park et al., 2014). Since autophagy has been shown to promote pancreatic cancer (Li et al., 2021), it is speculated that the L78 form of CHDH may not stimulate autophagy to inhibit pancreatic cancer.

#### Head and neck squamous cell carcinoma

As the sixth most common cancer, head and neck squamous cell carcinoma (HNSCC) is a malignancy of head and neck origin (Galizia et al., 2022). Wu et al. screened HNSCC patients for differentially expressed RNA based on the TCGA database and found that patients with HNSCC with low CHDH expression had the best prognosis using univariate Cox regression models (Wu et al., 2020).

#### Clear cell renal cell carcinoma

Clear cell renal cell carcinoma (ccRCC) is a prevalent and aggressive histologic subtype of renal cell carcinoma (RCC). Zhang et al. screened differentially expressed genes (DEGs) associated with clinical traits by DEG analysis and weighted gene co-expression network analysis (WGCNA), finally identified six prognostic metabolism-related genes including CHDH, and constructed a CHDH prognostic model, showing that low expression of CHDH was significantly associated with poorer overall survival (OS) in ccRCC patients. Based on the prediction of clinical chemotherapy response analysis, four drugs were screened, namely, Vinblastine, ZM.447,439, AP.24534 and CGP.60474, which may be more sensitive to patients in the experimental group in the high-risk group, which may be a potential treatment for ccRCC (Zhang et al., 2022).

#### Gastric cancer

With the popularity of gastrointestinal endoscopy, the cure rate of early gastric cancer (GC) has increased significantly, while the survival rate of patients with advanced GC remains low. The NF-κB family is a well-known family of transcription factors, and the NF-κB signaling pathway directly and indirectly controls key cancer markers (Capece et al., 2022). Bastian et al. analyzed the TCGA database of GC RNA-sequencing (RNA-seq) data of 27 NF-κB-related metabolic genes (NFMG) and included genes from CHDH. They then grouped GC patients by constructing a risk score (RS), and finally found that patients with lower CHDH expression in the high-risk group had poorer OS. Thus, the good performance of CHDH in predicting the OS of GC patients was validated (Chen et al., 2022).

#### Hepatocellular carcinoma

Hepatocellular carcinoma (HCC) is characterized by poor long-term prognosis and high mortality. Wei et al. constructed a protein-protein interaction network and identified CHDH as one of two targets in HCC tumor-adjacent noncancerous tissues. By comparing protein expression in tumors and adjacent tissues using IHC, they found that HCC patients with high CHDH expression in adjacent tissues had shorter recurrence-free survival and OS. This suggests that CHDH may be a promising biomarker and a potential therapeutic target for hepatocellular carcinoma (Wei et al., 2022).

### CHDH and male infertility

It is estimated that 8%–12% of couples worldwide are affected by infertility, with male infertility accounting for 50% of the total (Agarwal et al., 2021). The importance of CHDH in the male reproductive system has been demonstrated. Knockout of CHDH in male mice leads to altered mitochondrial morphology, reduced ATP content, and decreased sperm viability, resulting in reduced fertility in mice (Johnson et al., 2010). In a 2012 population-based study, the genotype and allele distribution of the CHDH +432G>T polymorphism differed significantly between oligozoospermic and normospermic males. The CHDH 432G/G genotype was associated with higher sperm concentration in oligospermic and normal men compared to the 432T/T genotype (Lazaros et al., 2012). In a study of male spermatozoa, Johnson et al. suggested that the CHDH rs12676 (+432G>T) SNP was associated with abnormal mitochondrial ultrastructure, reduced ATP concentration in spermatozoa and altered sperm motility patterns (Johnson et al., 2012). SNP-induced changes in CHDH activity may lead to male infertility.

### CHDH and hyperhomocysteinaemia

Abnormal accumulation of homocysteine leads to hyperhomocysteinemia, which is an independent risk factor for cardiovascular disease (Li et al., 2019). CHDH deficiency leads to elevated homocysteine in the blood because betaine is one of the major methyl donors for the conversion of homocysteine to methionine (Ohnishi et al., 2019). Kumar et al. proposed that the CHDH rs9001 (+119A>C) SNP polymorphism is associated with homocysteine levels. Individuals with the AA genotype had higher homocysteine levels than those with the CC genotype. As the main organ of CHDH expression, the liver is also the main site of homocysteine metabolism, so the CHDH polymorphism may play a key role in maintaining circulating homocysteine levels (Kumar et al., 2009).

### CHDH and metabolic syndrome

CHDH is one of the key enzymes in choline metabolism and SNPs in CHDH gene affect the choline metabolic pathway (Da et al., 2006). It was found that rather than generating betaine, women with the CHDH rs9001 variant allocated more choline to the CDP-choline pathway after choline intake. In addition, women with the CHDH rs12676 variant seem to prefer dietary choline for PEMT-PC synthesis (Ganz et al., 2017). The study by Konstantinova et al. monitored the relationship between levels of choline and its metabolite betaine in the blood and components of metabolic syndrome, such as body fat percentage, blood pressure, serum lipid, etc. High levels of choline and low levels of betaine in the blood were found to be positively associated with a higher risk of cardiovascular disease, whereas the amount of choline and glycine betaine provided in the diet has no significant effect on the blood concentration of these compounds. The authors speculate that changes in blood levels of choline and betaine may be due to mitochondrial biosynthesis dysfunction of betaine, rather than dietary patterns (Konstantinova et al., 2008).

### CHDH and bipolar disorder

Bipolar disorder (BD) is a psychiatric disorder with complex clinical manifestations ranging from depressive episodes to manic episodes. In a study by Chang et al. mRNA expression of the choline dehydrogenase gene was upregulated in the brains of BD patients compared to healthy controls. Rs9836592, a genetic risk variant in a large sample group of the CHDH gene, was strongly associated with BD, and this allele was strongly associated with elevated CHDH in the human brain. Rs9836592 may be associated with dysfunction of the regulatory component of CHDH that affects CHDH mRNA expression, leading to BD (Chang et al., 2017). Trang et al. showed a significant dose-dependent downregulation of CHDH after treatment of NT2-N cells with lamotrigine and quetiapine compared to dead brain cells from BD patients (Truong et al., 2022). This provides a new theoretical basis for the pharmacological treatment of patients with BD.

### CHDH and tooth agenesis

Tooth loss is one of the most common anomalies of human teeth. Polymorphisms in genes of the folate and choline metabolic pathways may induce susceptibility to cleft lip and/or cleft palate (CL/P) in different populations (Murthy and Bhaskar, 2009). Adrianna et al. investigated the association between SNPs of genes related to choline metabolism and tooth loss and found that individuals with the CHDH rs6445606 C allele had a 2-fold reduced risk of tooth loss. Individuals with one or two rs6445606 C alleles had a differentially reduced risk of tooth loss compared to carriers of the rs6445606 T allele (Mostowska et al., 2011). This suggests that SNPs of the CHDH gene are closely associated with tooth agenesis.

### CHDH and down syndrome

Down syndrome (DS) is caused by chromosome nondisjunction in meiosis I stage. Impairment of folate metabolism can hypermethylation of DNA around chromosomes, leading to nondisjunction of chromosome 21. The association between genetic polymorphisms in the folate-homocysteine metabolic pathway and the risk of DS has been demonstrated (Sukla et al., 2015). By genotyping all SNPs of mothers whose children had Down syndrome or not, Jaiswal et al. found no significant differences in CHDH rs12676 allele and genotype frequencies. However, the cholinesterase genotype combination PEMT - 744GC/CHDH +432GG/BHMT +742GG was significantly higher in mothers of children with Down syndrome (Jaiswal et al., 2017). Thus, impaired choline metabolism is closely associated with DS.

### CHDH and schizophrenia

Current treatments for psychiatric disorders rely on monoamines, and there is an urgent need to discover novel therapeutic agents. Ohnishi et al. found that deficiency of CHDH activity in mice reduced betaine production, leading to psychobehavioral and schizophrenia related molecular changes in the brain. In a pharmacological animal model of schizophrenia, betaine supplementation corrected the altered antioxidant and pro-inflammatory responses characteristic of this model. The expression levels of CHDH rs35518479 in postmortem brains of schizophrenia patients were found to be significantly different from normal controls by eQTL analysis. CHDH expression was significantly higher in the brains of A allele carriers compared to G/G homozygotes. Patients with the A allele had a higher SAM/SAH ratio (methylation index), implying that CHDH expression levels affect the turnover rate of the methionine-homocysteine cycle. Moreover, depressive features were found in CHDH-deficient mice in this study (Ohnishi et al., 2019).

## Detection of CHDH

CHDH, a specific enzyme in the mitochondrial membrane, affects human growth and development by participating in choline metabolism and mitochondrial autophagy. When choline intake is adequate, deficiency or alteration of CHDH activity may be the underlying cause of the associated diseases. SNPs of CHDH genes are closely associated with the development of diseases, and SNPs have the potential to lead to altered CHDH activity (Table 2). There are few studies focus on the signaling pathway of CHDH, only Chen Q et al. performed bioinformatics analysis and demonstrated that CHDH may be an NF-κB-related metabolic gene (NFMG) (Chen et al., 2022). Although some studies mentioned that betaine as an endogenous agonist of AMPK which regulates metabolic processes, this did not mean that CHDH is directly involved in this signaling pathway (Li et al., 2017; Chen et al., 2021). During the mitochondrial autophagy function of CHDH, researchers did not find any cleavage or post-translational modifications of CHDH, such as ubiquitination (Park et al., 2014). For the detection of CHDH, a spectrophotometric method has been proposed in 1950s to detect choline dehydrogenase activity in rat liver and kidney homogenates (EICHEL, 1954). Moreover, CHDH proteins of mouse, rat and human origin can be detected by Western blot, immunoprecipitation, immunofluorescence and Enzyme linked immunosorbent assay. In addition, synthetic CHDH primers using cytochrome b (MT-CYB) and ACTB can be used for PCR analysis of extracted DNAs (Park et al., 2014). Interestingly, high-resolution melting (HRM) can be used for molecular diagnosis of CHDH gene SNP loci in diseased tissue samples, providing a new method for clinical diagnosis and prognosis prediction of diseases through routine and low-cost molecular diagnosis.

**TABLE 2:** Summary of CHDH SNPs correlated with corresponding disease.

dpSNP ID	Allele	Phenotype	Disease	Ref.
rs12676	432G>T	L78R	Breast cancer	Xu et al. (2008)
			Pancreatic cancer	Chittiboyina et al. (2018)
			male infertility	Johnson et al. (2012)
rs9001	119A>C	E40A	hyperhomocysteinaemia	Kumar et al. (2009)
rs9836592	C/G, T	Intron	bipolar disorder	Chang et al. (2017)
rs6445606	C/G, T	Intron	tooth agenesis	Mostowska et al. (2011)
rs35518479	A>G	Intron	schizophrenia	Ohnishi et al. (2019)

## Discussion and future prospects

Recent studies have found that the expression of CHDH varies in different tumor types and that CHDH is closely related to biological processes such as tumor proliferation and prognosis, but a method to detect CHDH as a tumor marker in serum has not been mentioned (Roci et al., 2020; Kuai et al., 2023). Based on this, it is expected to clarify the expression of CHDH in different tumors and discover the assay of CHDH as a tumor marker in serum. Moreover, as a result of lacking of stable, active and highly purified enzymes, little research on the biochemical characteristics of CHDH has been performed. It is expected to purify CHDH and clarify the biochemical characteristics of the enzyme, thus providing a theoretical basis for the polymorphism of CHDH gene leading to altered enzyme activity. In addition, based on the resolution of CHDH structure, the design and synthesis of specific small molecule inhibitors or activators of CHDH may provide new targets and therapeutic strategies for metabolic disorders diseases as well as tumor.
